# Cardiovascular disease and radiopharmaceutical therapies– an underestimated risk?

**DOI:** 10.1007/s00259-024-07039-4

**Published:** 2025-01-02

**Authors:** Michael Lassmann, Uta Eberlein, Frederik A. Verburg

**Affiliations:** 1 Department of Nuclear Medicine, University Hospital Würzburg, D-97080 Würzburg, Germany; 2 Department of Radiology and Nuclear Medicine, Erasmus MC, Rotterdam, 3015 GD The Netherlands

**Keywords:** Cardiovascular disease, Radiopharmaceutical therapy, Absorbed dose

Cardiovascular disease (CVD) is one of the major causes of death world-wide [1]. The fact that ionizing radiation can damage the cardiovascular system has been emphasized in a meta-analysis on the relationship between ionizing radiation and CVD by Little et al. [2] and in a recent review by Jahng et al. [3]. These two publications summarize the evidence on the occurrence of cardiovascular disease (CVD) from radiation exposure due to radiation accidents, occupational exposure, and external beam radiation therapy.

A few years ago, the International Commission on Radiological Protection (ICRP) established its Task Group 119 (“Effects of Ionising Radiation on Diseases of the Circulatory System and their Consideration in the System of Radiological Protection”) with the aim to review the recent scientific literature related to radio-epidemiological and radiobiological studies of CVD. The aim of the task group is to provide advice on the evidence, available on risk of CVD over all dose levels, including considerations concerning a dose-threshold. The final goal is to provide advice on how to reflect current knowledge of radiogenic CVD in the system of radiological protection.

In the meta-analysis by Little et al. [2], a significant excess relative risk per Gy was noted for exposures of less than 0.5 Gy. The authors state that “findings from certain cohorts with occupational exposure to radiation (such as the Mayak workers) strongly suggest a substantial contribution of internal emitters to cardiovascular risk, independent of the externally received dose. However, a clear association between internal emitters and cardiovascular disease has not been reported elsewhere.” [2]. This raises the question whether internal exposure by radiopharmaceuticals also increases the risk for CVD.

Concerning the absorbed doses to the cardiovascular system, Kolbert et al. showed for 26 thyroid cancer patients [4] that the absorbed dose to the blood is a potential surrogate for the absorbed dose to the cardiovascular system. For radiopharmaceutical therapies, several studies observed absorbed doses to the blood of at least 0.1 Gy per treatment cycle, resulting in cumulated absorbed doses of more than 0.3 Gy after several cycles of treatment [4–7]. Combining these clinical data with the results of the meta-analysis by Little et al. [2] leads to the hypothesis that radiopharmaceutical therapies increase the long-term risk for CVD. However, data for radiopharmaceutical therapies are sparse, as of today only few epidemiological studies report on the risk for CVD after radioiodine therapy (RAI) of thyroid cancer [8–10].

Klein Hesselink et al. reported results of an observational study on long-term cardiovascular mortality [8]. The median administered activity of ^131^I for their patient cohort was 7.4 GBq with a median follow-up of 10.5 years. This study was the first to report an increased risk of cardiovascular and all-cause mortality in patients with DTC, independent of age, sex, and cardiovascular risk factors [8]. However, in a model also accounting for the significant influence of median TSH level, the cumulative ^131^I activity was not identified as a significant predictor.

Another study by Kao et al. [9] demonstrated that RAI was not associated with a higher risk of CVD or CVD-specific mortality compared to non-RAI cases, including that of specific disease subgroups. However, they found that a cumulative administered activity of more than 3.7 GBq was associated with a higher risk of developing CVD, and the cumulated administered activity of more than 5.55 GBq was associated with a higher risk of CVD-specific mortalities [9].

Kim et al. [10] studied the effects of radioiodine therapy on cardiovascular disease in South Korean thyroid cancer patients. In their patient cohort, the median cumulative administered activity was 3.7 GBq. Their study found no significant between-group difference for the CVD risk in patients with thyroid cancer who received RAI and those who did not, however, their median follow-up of 5.5 years was shorter compared to the studies of Klein Hesselink et al. [8] and Kao et al. [9].

For thyroid cancer, we could show in a group of 209 patients with differentiated thyroid cancer that the absorbed dose to the blood is (a) a better predictor of success of RAI, and (b) that there is a high variability in the absorbed dose to the blood for patients receiving the same administered activity [5]. In this study on patients receiving RAI with the intent of either remnant ablation or adjuvant treatment, the median absorbed dose to the blood was 0.3 Gy. If patients receive more than one course of RAI, there is a high likelihood that the absorbed dose to the blood and, consequently, the cumulated absorbed dose to the cardiovascular system exceeds 0.5 Gy. It also indicates that, considering the wide spread of the correlation between activity and absorbed dose to the blood, the results of the three studies cited above [8–10] potentially mask absorbed dose effect relationships by reporting the administered activity only.

For prostate cancer therapy with [^177^Lu]Lu-PSMA, Schumann et al. reported a median absorbed dose to the blood of 0.1 Gy for a single treatment cycle for an activity of 6 GBq [^177^Lu]Lu-PSMA I&T [7]. For [^177^Lu]Lu-PSMA-617 a recalculation of the published data of the dosimetry study of the VISION trial [11] results in an estimated absorbed dose to the blood of about 0.3 Gy for one cycle with 7.4 GBq [^177^Lu]Lu-PSMA-617. Since for prostate cancer treatments six treatments cycles are foreseen, in many patients resulting absorbed doses to the blood of more than 0.5 Gy can be expected. As the life expectancy of the patients who are currently treated with [^177^Lu]Lu-PSMA is limited, no effects on the CVD are likely to be observed. However, if [^177^Lu]Lu-PSMA therapy is applied at an earlier stage of the disease, the risk of developing CVD may become non-negligible.

In conclusion, although a clear relationship between radiation exposure from radiopharmaceutical therapy and CVD has not yet been established with scientific certainty, there is sufficient evidence in the literature to suggest that such a relationship may exist, and caution is warranted. We therefore advocate that the absorbed doses to the cardiovascular system after radiopharmaceutical therapies be closely monitored in order to obtain reliable data on the relationship between the absorbed doses and CVD. This in turn might help to better define the risk factor for CVD after the use of ionizing radiation.
